# Analysis of vertically loaded piles considering crushing characteristics of crushed stones

**DOI:** 10.1371/journal.pone.0219003

**Published:** 2019-07-11

**Authors:** Chong Jiang, Yu Li, Jia-li He

**Affiliations:** School of Resources and Safety Engineering, Central South University, Changsha, Hunan, China; China University of Mining and Technology, CHINA

## Abstract

In this paper, a method was developed to assess the vertical response of piles considering crushing characteristics. A load transfer model of a pile is built and the relationship between the crushing characteristics and load transfer function of a pile was analyzed. Based on the analysis results, the transfer equation of load function considering the crushing characteristics was derived using the finite difference method. The ideal side resistance and the load transfer mechanism of a pile can be obtained by the transfer equation of load function. The predicted response of piles with the proposed method is in good agreement with the response observed in some pile tests reported in the literature. Further study was conducted to discuss the effect of the parameters related to the load transfer model considering crushing characteristics on the load-settlement response of a single pile.

## Introduction

Karst caves are inevitably encountered during tunnel construction in western China. In order to strengthen the bearing capacity of rock-socketed pile foundations in the karst cave below the tunnel, the rock-soil aggregate from excavation is backfilled around the rock-socketed pile foundations. However, the effect of backfill is ignored in engineering practice because the design is too conservative. It was found that the pile side resistance from field tests bore most of the total load and increased with the increase of the pile modulus ratio [1]. Therefore, it is of great significance to further study the effect of backfill on the bearing mechanism of the pile foundation.

In past decades, a number of methods have been developed for pile response analysis, included the theoretical load transfer curve method [2–7], the shear displacement method [8–12], finite element method [13–16], the boundary-element method [17–20], the variational approach [21–24] and the field test [25–27], etc. It is worth mentioning that these studies reported that the pile was surrounded by clay, sand or rock-soil aggregate. However, the pile foundation of a tunnel is surrounded by crushed stones in many cases, especially in the case of backfilling. The load—settlement curve of backfill piles in the large karst area is developed by Jiang et al. [28]. Chen et al. [29] proposed a method for the calculation of the load settlement of karst cave backfill pile foundations under low confining pressure. In the process of backfilling, crushed stones will be rolled to make it more compact. However, some research [28–29] failed to consider that crushing is often caused by a rolling load in the backfilling process, and the crushing has great influence on the mechanical properties of crushed stones [30–33]. Therefore, more extensive studies are warranted to assess the vertical response of piles considering crushing characteristics.

In light of this, this study investigates the relationship between the crushing characteristics of crushed stones and the load transfer of the pile foundation, and then analyzes the load transfer mechanism of piles caused by crushing characteristics. The effect of crushing characteristics on the load transfer response of the pile surrounded by crushed stones is also discussed. The results calculated by the proposed method in this paper are compared with the measured results from other research.

## Establishment of the pile foundation model in crushed stones backfill

### Establishment of the transfer function and assumptions

**Fig 1:**
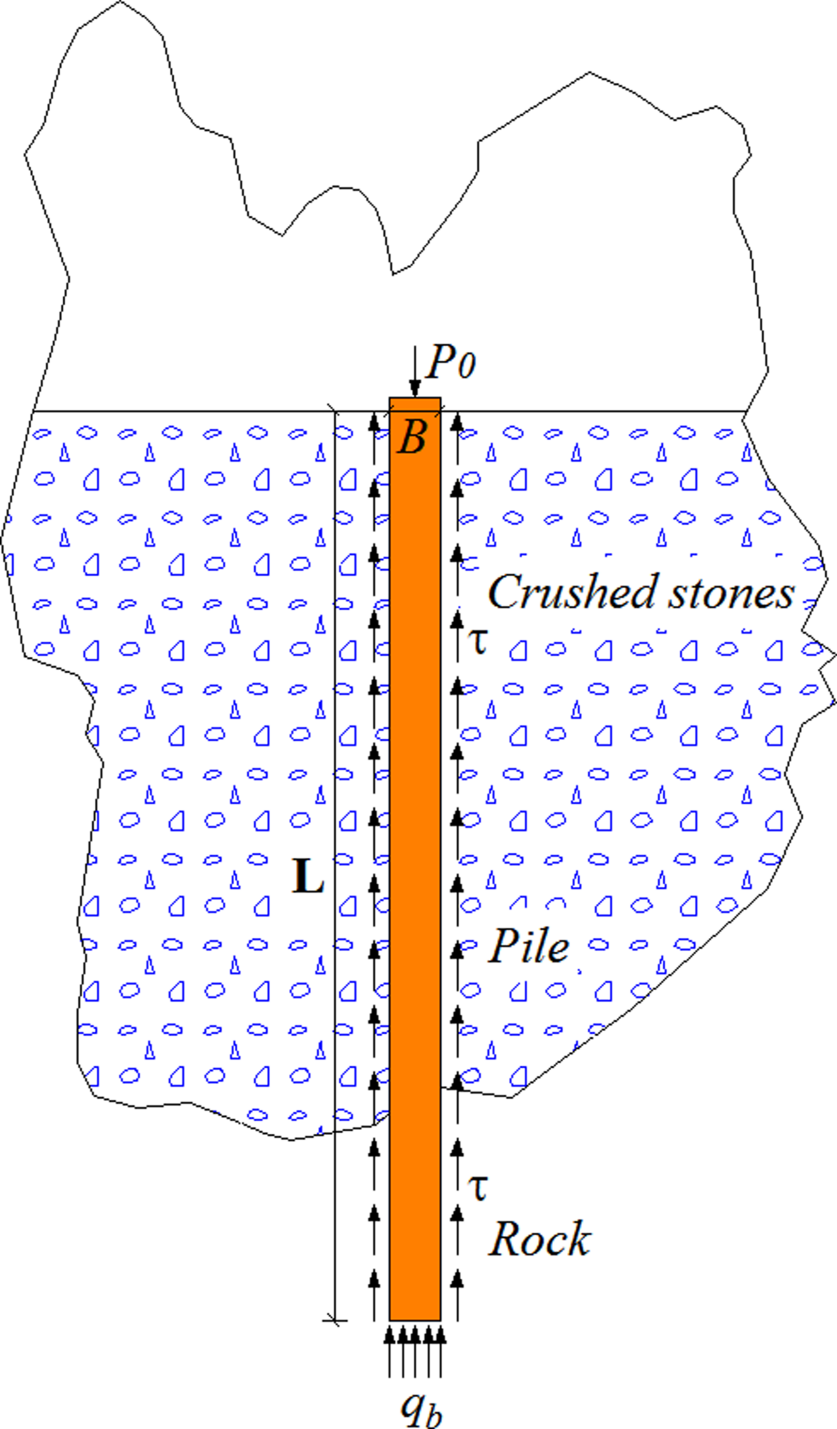
Geometry of a pile in crushed stones.

**Fig 2:**
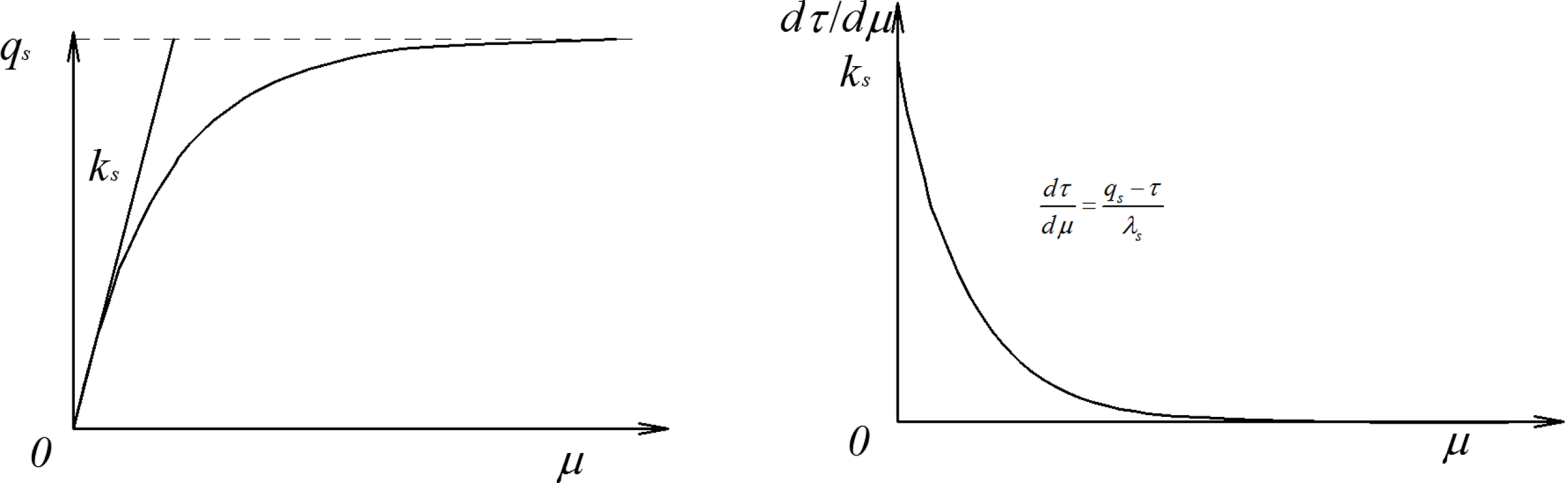
Nonlinear variation of soil stiffness around the pile.

Generally, researchers study the vertical response of piles using transfer function method, shear displacement method, elastic theory method and numerical method. The primary difference in various methods is the assumption of the pile-soil model. The transfer function method is simple for engineering practice. In fact, the key to the calculation method of rock-socketed pile foundations in crushed stones backfill area (Fig 1) is the selection of the transfer function. A linear model was usually used to calculate the settlement of vertically loaded pile. However, the measured results in some engineering practice show that the load transfer function curves of pile side were generally nonlinear [2, 34, 35]. Subsequently, an exponential model was built to simulate the nonlinear variation of the pile side resistance. The stiffness of the soil around the pile was assumed in the ‘AB1’ model [7] (Fig 2 and Eq (1)).

$$\frac{d\tau }{d\mu }=\frac{q_{s}-\tau }{\lambda_{s}}$$ (1)

In a recent study, the complexity associated with characteristics of crushed stones was not thoroughly studied, and the flaw rested with the mobilization of the pile side resistance for crushed stones [28]. It is worthwhile to build a load transfer model considering breakage of crushed stones around the pile. The side resistance and the base resistance is obtained by the integration of Eq (1).

$${\tau (\mu )=q}_{s}\left({\text{1-e}}^{\text{-}\frac{\mu }{\lambda_{\text{s}}}}\right)$$ (2)

$${\text{q}(\mu )}_{b}{=q}_{b}\left({\text{1-e}}^{\text{-}\frac{\mu_{b}}{\lambda_{\text{b}}}}\right)$$ (3)

where *q*_*s*_, *q*_*b*_, *μ* and *μ*_*b*_ are, respectively, the ultimate side resistance, the ultimate base resistance, the axial displacement and the base axial displacement. *λs* and *λ*_*b*_ are the parameters taking into account the effect of the crushed stones type and rock type, respectively. *t*–*z* curves come from the function *τ*(*μ*) which describes the variation of the mobilized side resistance *τ* in the function of the axial displacement *μ*. And the function *q*(*μ*_*b*_) describing the variation of the mobilized base resistance *q* in the function of the base axial displacement *μ*_*b*_ is obtained.

$$\lambda_{s}=\frac{Bq_{s}}{\alpha_{s}E_{M}}$$ (4)

$$\lambda_{b}=\frac{Bq_{b}}{\alpha_{b}E_{M}}$$ (5)

Where *B* is the pile diameter, *E*_*M*_ is the Ménard modulus, *α*_*s*_ and *α*_*b*_ is the factors taking into account the effect of the soil type. *α*_*s*_ is 2.0 and *α*_*b*_ is 9.0 in chalk and crushed stones [7, 28]. When *μ* = 0 and *μ*_*b*_ = 0, *k*_*s*_ and *k*_*b*_ are the initial stiffness of the pile side and pile base respectively.

$$\frac{d\tau }{d\mu }\left(0\right)=\frac{q_{s}}{\lambda_{s}}=\frac{\alpha_{s}E_{M}}{B}=k_{s}$$ (6)

$$\frac{d\tau }{d\mu_{b}}\left(0\right)=\frac{q_{b}}{\lambda_{b}}=\frac{\alpha_{b}E_{M}}{B}=k_{b}$$ (7)

**Fig 3:**
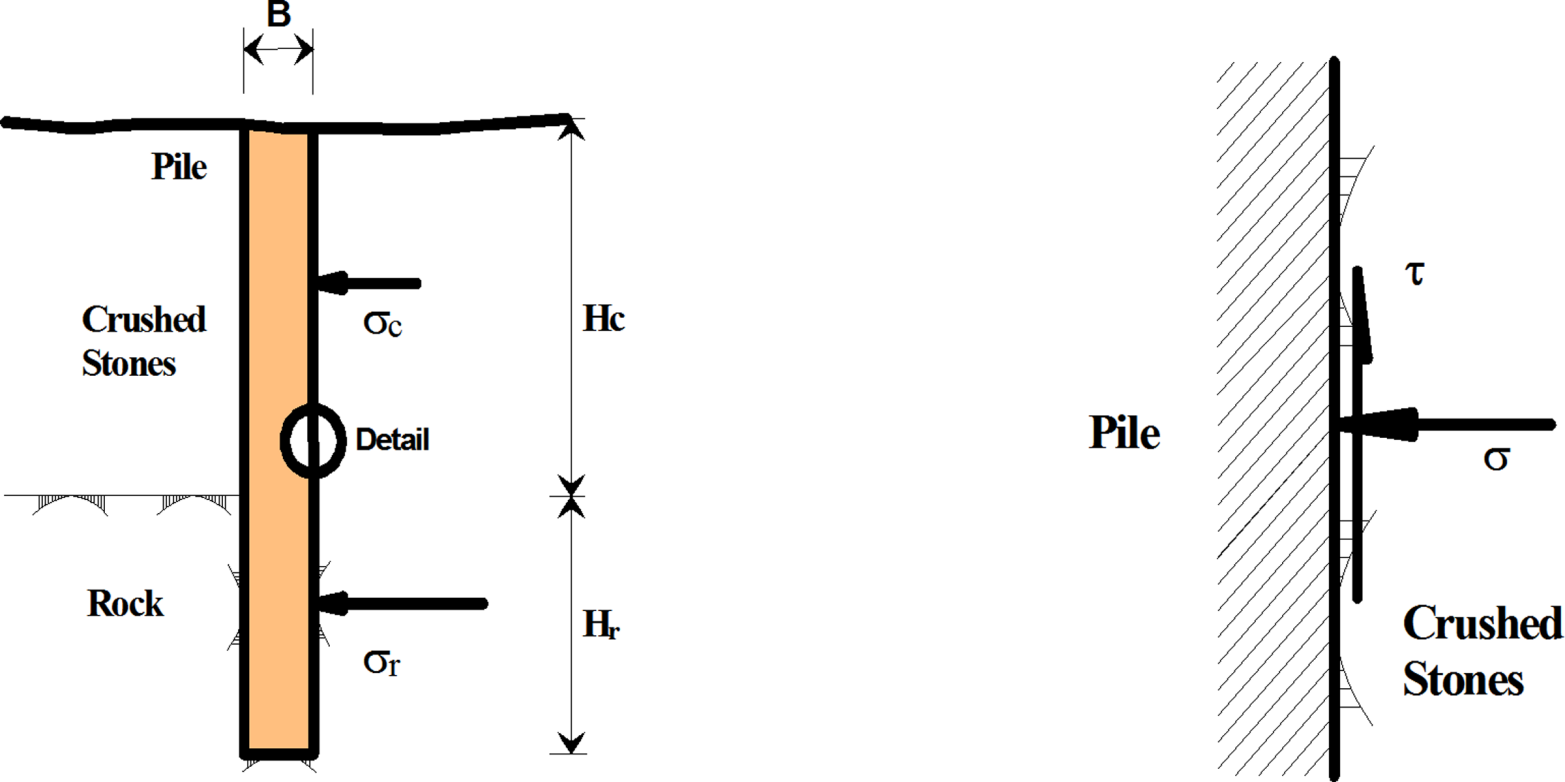
Schematic diagram of stresses applied on a pile.

The morphology and the geometrical conditions that are assumed for the pile in backfill area and the detail stresses diagram in the pile side are shown in Fig 3. *B* is the pile diameter. *σ*_*3*_ is the rolling load and *τ* is the mobilized side resistance. *σ*_*c*_ and *σ*_*r*_ are the horizontal stresses that are applied on the pile in the crushed stones and rock, respectively. *H*_*c*_ and *H*_*r*_ are the depth of the pile in the crushed stones and rock, respectively.

To formulate pile foundation model in backfill area considering the crushing characteristics, in terms of the interaction between the pile and crushed stones, assumptions and statements are made in advance.

1. In the same horizontal plane of pile, the settlement of the crushed stones backfill is the same, and the corresponding displacement is the same. There is only vertical displacement for a pile under vertical loading and the radial displacements for a pile is negligible.
2. The crushed stones and rock around the pile are considered as a series of independent nonlinear springs, and the pile is modeled as an elastic-plastic material.
3. The peak internal friction angle of crushed stones is assumed to be the internal friction angle of the Mohr-Coulomb criterion. Before the crushed stones are backfilled, the rolling load is equivalent to the confining pressure of the triaxial test of compacted crushed stones.

**Fig 4:**
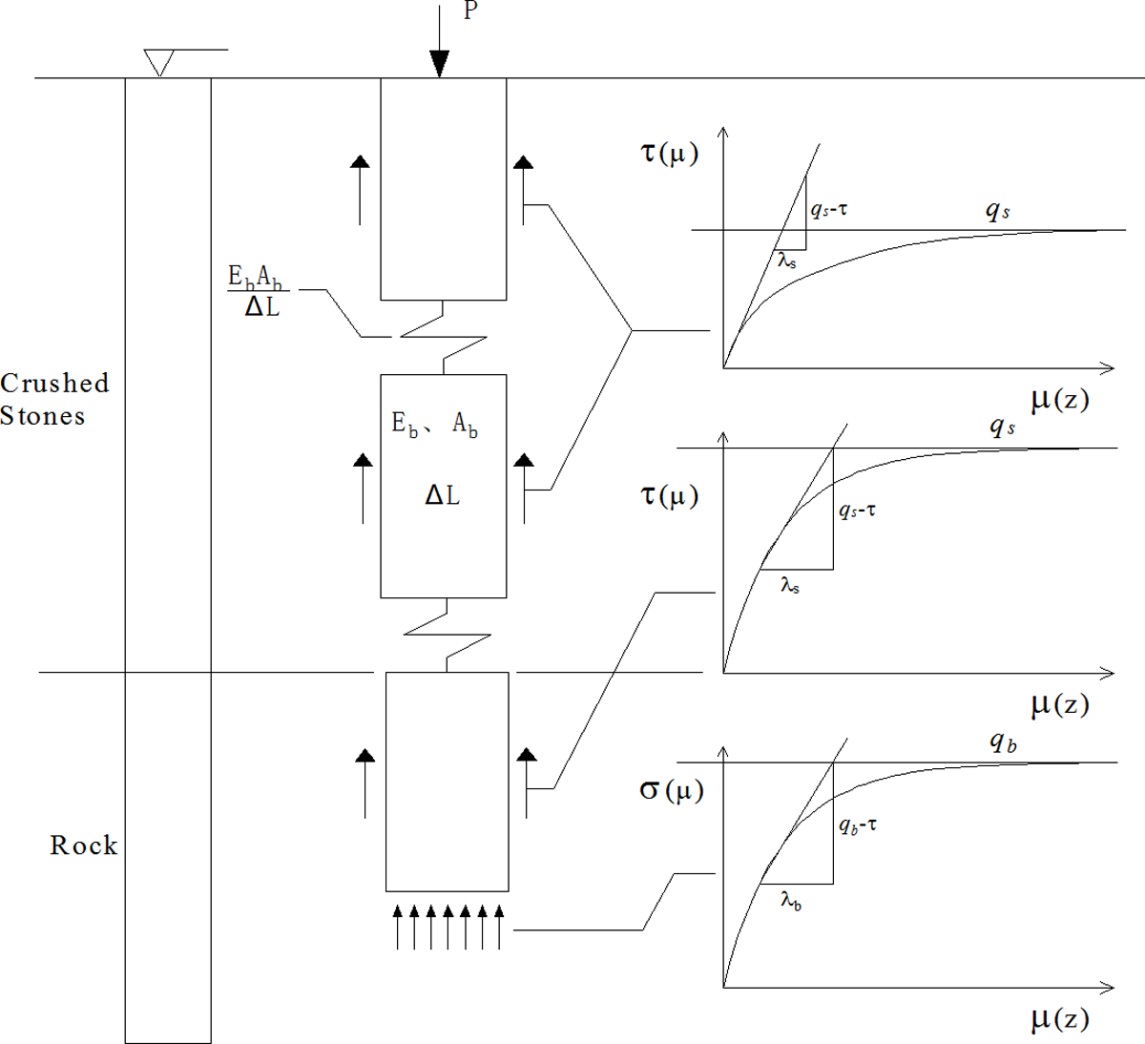
Analytical model of a socketed pile in backfill area.

Based on the above assumptions, the relationship between the function *τ*(*μ*) of the crushed stones, rock of pile side, and the function *q*(*μ*_*b*_) of rock of the pile base and corresponding displacement is shown in Fig 4.

### Relative breakage

Up to now, breakage often takes place when crushed stones are compressed or sheared [36–37]. The breakage (*B*_*g*_) proposed by Marsal [38] is the most commonly used parameter in particle breakage. Bai [39] proposed *B*_*60*_ to estimate crushing characteristics by measuring the particle size difference before and after loading. The relative breakage *B*_*r*_, proposed by Hardin [30], is stable and can also reflect the size variation of each particle before and after the test. Therefore, the relative breakage *B*_*r*_ of crushed stones is used to measure the crushing characteristics.

$$B_{r}=\frac{\int_{0}^{1}\left(b_{p0}-b_{pl}\right)df}{\int_{0}^{1}b_{p}df}$$ (8)

Where *b*_*p*_ represents the potential for breakage that is significant to soil behavior; *df* is a differential of “percent passing” divided by 100; *b*_*p0*_ is the original values of *b*_*p*_; *b*_*pl*_ is the original values of *b*_*p*_ after loading.

Crushed stones compaction is warranted to improve the pile bearing capacity. The effect of rolling loads on the crushed stones breakage depends on the types of rolling load. Based on the crushing test of equal-pressure consolidation particles under different confining pressure by large Triaxial tester, the relationship between breakage *B*_*r*_ and the rolling load *σ*_*3*_ was obtained [31]. The $\frac{\sigma_{3}}{p_{a}}/B_{r}-\frac{\sigma_{3}}{p_{a}}$ plots show a good linear relationship. By fitting the formula of the linear relationship, the relationship between the relative breakage *B*_*r*_ and the rolling load *σ*_*3*_ are given:

$$B_{r}=\frac{\sigma_{3}/p_{a}}{a+b\left(\sigma_{3}/p_{a}\right)}$$ (9)

Where *a*, *b* are fitting parameters, which are obtained by fitting the test results of $\frac{\sigma_{3}}{p_{a}}/B_{r}-\frac{\sigma_{3}}{p_{a}}$, and *P*_*a*_ is atmospheric pressure to convert relative breakage *B*_*r*_ into dimensionless quantity.

Based on the fitting experimental data, the relationship between the peak internal friction angle *φ*_*p*_ and the relative breakage *B*_*r*_ was obtained by Indraratna [32]:

$$\varphi_{p}=j{\left(B_{r}\right)}^{k}$$ (10)

Where *j* and *k* are test parameters of the particle. The fitting result is shown in Table 1 [31]:

**Table 1:** Fitting coefficients for various types of rockfill.

Samples	Lithology	ρ/g·cm^-3^	a	b	R^2^	j	k	R^2^
1	Limestone of Mao-kou formation	2.10	0.271	0.073	0.987	85.285	-0.321	0.995
2	Limestone	2.07	0.523	0.061	0.941	81.138	-0.277	0.970
3	Dolomitic limestone	2.03	0.476	0.086	0.956	58.867	-0.135	0.990
4	Dolomitic limestone	2.03	0.551	0.053	0.987	61.821	-0.168	0.996
5	Dolomitic limestone	2.03	1.051	0.098	0.946	56.208	-0.157	0.982
6	Quartz sandstone	2.12	0.209	0.043	0.999	65.232	-0.177	0.967
7	Limestone of Mao-kou formation	2.15	0.373	0.039	0.990	59.088	-0.132	0.943
8	Limestone of Xi-xia formation	2.10	0.264	0.051	0.986	59.553	-0.153	0.922

### Ultimate side resistance

**Fig 5:**
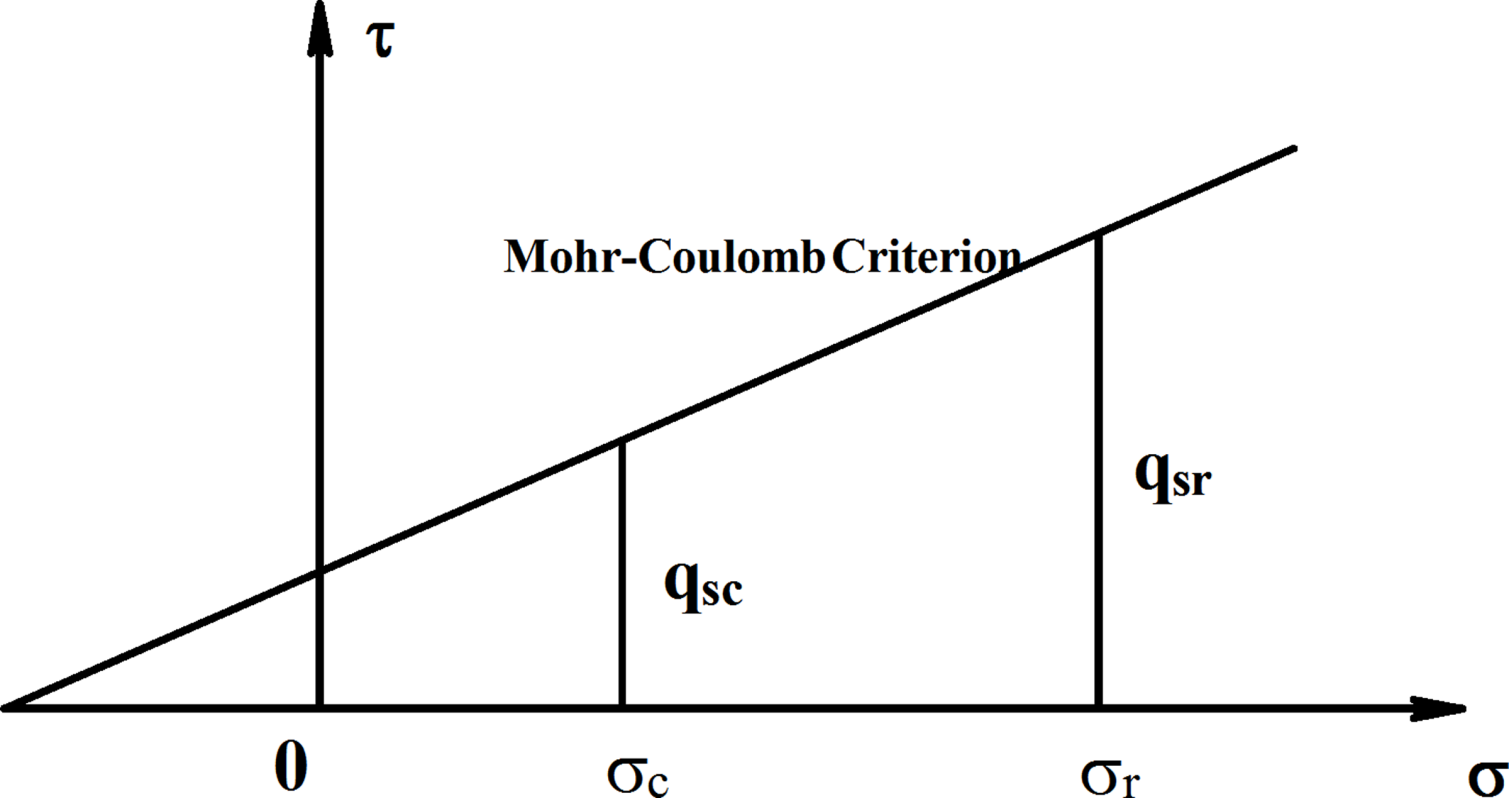
Mohr-Coulomb failure criterion.

The Eqs 9 and 10 show that the rolling load and the relative breakage affect the internal friction angle of crushed stones. The internal friction angle is related to the ultimate side resistance of crushed stones. Thus, it is necessary to analyze the effect of relative breakage on the ultimate side resistance. Triaxial tests on rock particles were carried out to determine the strength characteristics of the particle under different horizontal stress [33]. The cohesion, internal friction angles, Mohr’s circle and the shear strength envelope of the samples under different horizontal stress are in good agreement with the Mohr-Coulomb strength calculation formula, as shown in Fig 5.

$$q_{s}=c+\sigma \text{tan}\varphi_{p}$$ (11)

Where *c*, *σ* and *φ*_*p*_ are the cohesion of crushed stones, the horizontal stress and the peak internal friction angle of crushed stones, respectively.

The shear plane occurs on the vertical plane and the horizontal stresses on the pile side varies with depth. Therefore, the horizontal stress can be expressed as Eq (12).

$$\sigma =k_{0}\gamma z$$ (12)

Where *k*_*0*_, *γ*and *z* are the lateral pressure coefficient, the crushed stones bulk density and calculated point depth, respectively; *q*_*sc*_ and *q*_*sr*_ are the ultimate side resistance at the horizontal stress *σ*_*c*_ and the ultimate side resistance at the horizontal stress *σ*_*r*_, respectively.

## Analysis of vertical load transfer

### Governing equation and solution

**Fig 6:**
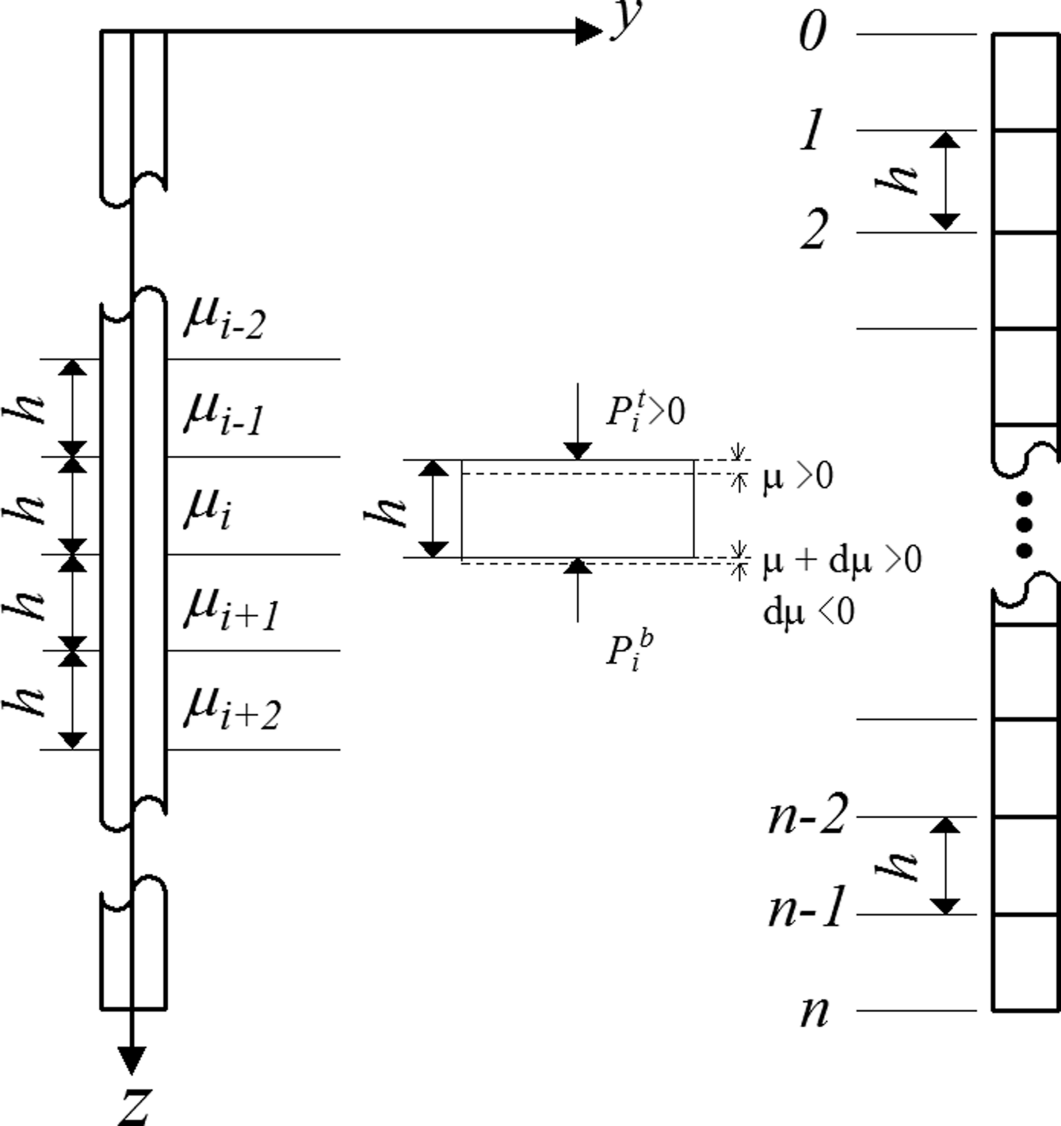
Diagram of the finite difference method.

As shown in Fig 6, the whole length *L* of pile is divided into *n* segments by the finite difference method. Each segment is *h* = *L*/*n*. The node of pile top and pile base are denoted as 0 and *n*, respectively. The *i*-th segment is subjected to the side resistance *τ* along the pile. The force of the upper segment and the lower segment are represented by *P*_*i*_^*t*^ and *P*_*i*_^*b*^, respectively. Based on the force equilibrium of the circular pile segment located at depth *z*, Eq (13) is obtained:

$$E_{i}A_{i}\frac{d^{2}\mu }{dz^{2}}-U\tau (\mu )=0$$ (13)

Where *E*_*i*_, *U*, and *A*_*i*_ are the Young’s modulus, the circumference and the segment area of the pile, respectively. Eq (14) is obtained by substituting the load transfer function Eq (2) and the ultimate side resistance expression Eq (11) into Eq (13).

$$\frac{d^{2}\mu }{dz^{2}}=\frac{U}{E_{i}A_{i}}\left({\text{1-e}}^{\text{-}\frac{\mu }{\lambda \text{s}}}\right)(\text{c}+{\sigma \text{tan}\varphi }_{p})$$ (14)

In this model, the boundary conditions between each segment are as follows:

$$\{\begin{array}{l} \mu_{\text{i}}^{b}=\mu_{\text{i+1}}^{\text{t}}\\ E_{i}A_{i}\mu^{'}{{}_{i}}^{b}=E_{i+1}A_{i+1}\mu^{'}{{}_{i+1}}^{t} \end{array}$$ (15)

Where b and t correspond to the base and top of the section, respectively; *i* is the *i*-th section; the displacement of the *i*-th node is *μ*_*i*_; $\mu^{'}{}_{i}$is the derivative of *μ*_*i*_.

According to the base axial displacement *μ*_*b*_, Eq (15) can be transformed into the two forms of boundary conditions as follows.

(1) *μ*_b_≤0

$$\left\{ \begin{array}{l} \mu_{1}^{\text{t}}=s_{0}\\ E_{1}A_{1}\mu^{'}{}_{1}=-P\\ \mu_{n}=0\\ E_{n}A_{n}\mu^{'}{}_{n}=0 \end{array}\right.$$ (16)

(2) *μ*_b_ >0

$$\left\{ \begin{array}{l} \mu_{1}^{\text{t}}=s_{0}\\ E_{1}A_{1}\mu^{'}{}_{1}=-P\\ E_{n}A_{n}\mu^{'}{}_{n}=A_{n}\text{q(}\mu_{n}\text{)} \end{array}\right.$$ (17)

Where *s*_*0*_ is the settlement of the pile top; *P* is the load applied on the pile top.

**Fig 7:**
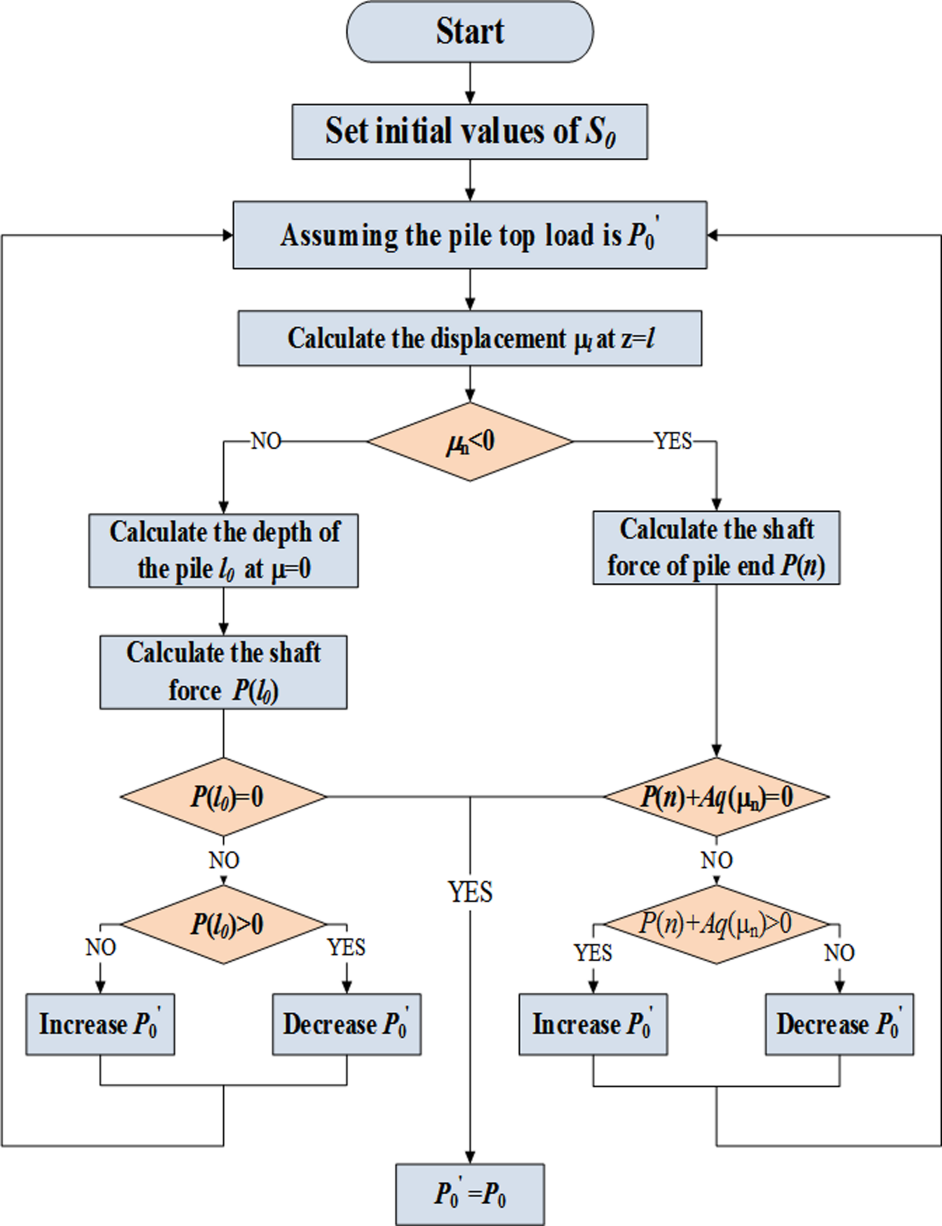
Program flow chart.

A program was written to compute the *Q*-*S* curves, side resistance and axial force according to boundary conditions. The program flowchart is shown in Fig 7.

### Comparison with other research

To validate the proposed model for the analysis of pile response, the proposed method in this paper is applied in this section to some pile load tests, which are well documented in the literature. The computed pile responses are compared to the pile test results. The pile tests considered are the tests reported by Carrubba [40] (Case 1) and Radhakrishnan [41] (Case 2).

**Fig 8:**
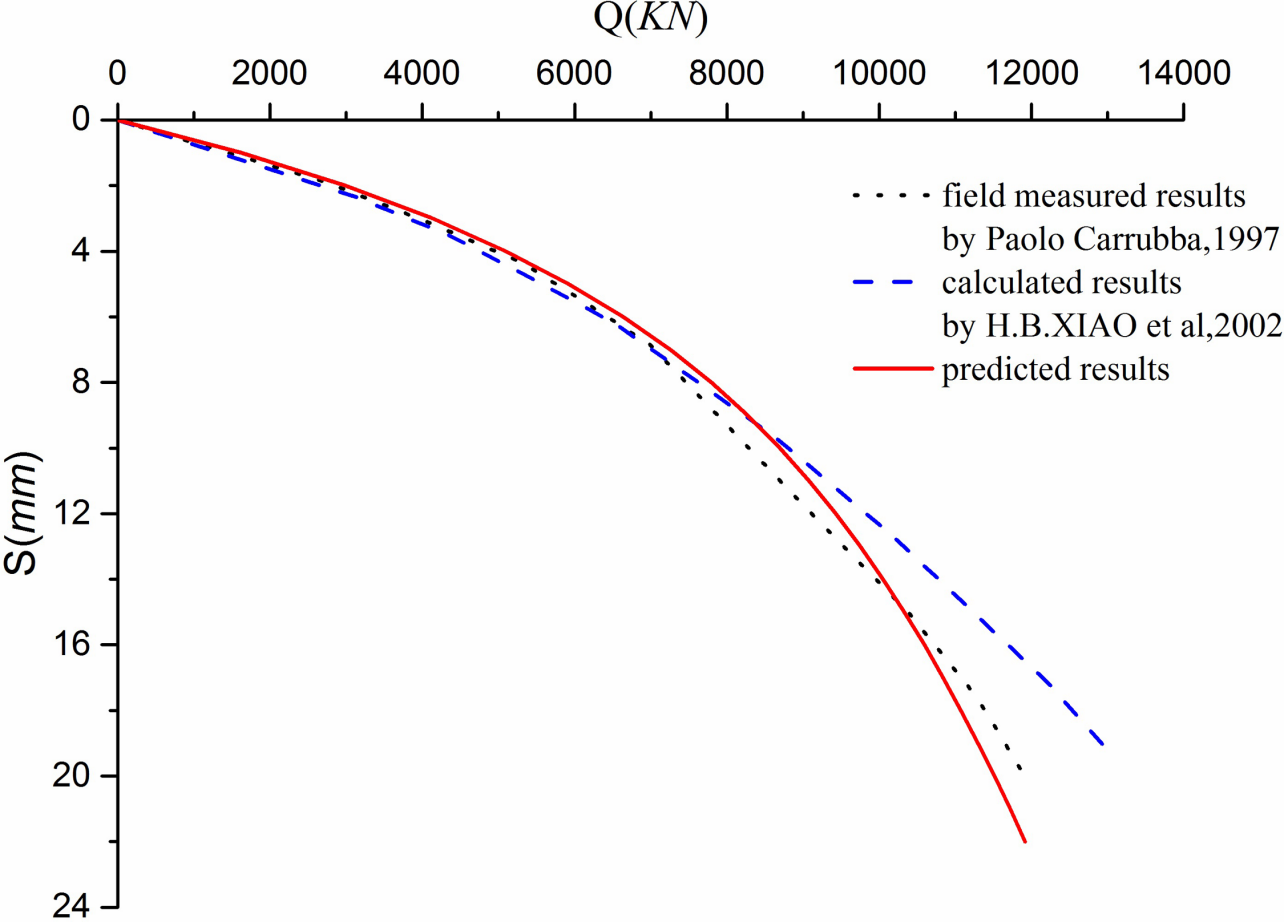
*Q*-*S* curves.

**Case1**. The first pile tests were performed to analyze the side resistance of large-diameter rock-socketed piles [40]. The pile diameter is 1.2 *m*; the pile length is 18.5 m; the pile depth in the granular soil and rock are 11*m* and 7.5 *m*, respectively; the Young’s modulus of the pile *E*_*b*_ is 31500*MPa*. It is difficult to determine the fitting parameters of breakage. Therefore, the value of *q*_*s*_ is used to verify the example. Based on the measured pile load-settlement curves, the back-calculated analysis and numerical method were used to determine the pile side resistance [42]. The parameters of the exponential load transfer function are summarized in Table 2. The pile responses calculated by the proposed method are compared to the pile test results [40] and to the pile responses computed by the method from Xiao [42], which is shown in Fig 8.

**Table 2:** Parameters of the exponential load transfer function in Case 1.

Soil layers	Siltstone properties	Location	Depth/*m*	*q*_*s*_ /*KPa*	*E*_*M*_ */MPa*	*α*_*s*_ */α*_*b*_	R^2^
1	Granular soil	Pile side	11.0	75	230	1.0	0.9373
2	Rock	Pile side	7.5	135	220	2.0	0.9625
2	Rock	Pile base	0	5000	300	9.0	0.8637

As illustrated in Fig 8, the methods in this paper and from Xiao [42] consider the nonlinear mobilization of side resistance. The results computed by the method from Xiao [42] are in good agreement with the measured results before the pile settlement of 9 mm while the results from the proposed method in this paper compare well with the measured results during the loading tests.

**Case 2**. In the pile tests conducted by Radhakrishnan [41], the pile TP1 was driven into highly weathered and fragmented siltstone. The pile diameter is 0.81*m* and the length of the pile is 11.5*m*. The unit shaft friction/shaft movement relationship with buried depth of 2m, 4m,8m and 10m is fitted by the parameters of the first, second, third and fourth layers, respectively. The parameters of the pile base are obtained by combing the figure of the variation of load versus depth with the load-settlement curve. The parameters of the exponential load transfer function are given in Table 3.

**Table 3:** Parameters of the exponential load transfer function in Case 2.

Soil layers	Siltstone properties	Location	Depth/*m*	*q*_*s*_ /*KPa*	*E*_*M*_ */MPa*	*α*_*s*_ */α*_*b*_	R^2^
1	dense sandy silt	Pile side	1.1	527	120	2.0	0.9083
2	medium dense heavily weathered & fragmented siltstone	Pile side	2.1	445	220	2.0	0.8661
3	medium hard fragmented siltstone	Pile side	6.6	226	77	2.0	0.9006
4	hard highly fractured siltstone	Pile side	1.7	196	40	2.0	0.7521
4	hard highly fractured siltstone	Pile base	0	1000	60	9.0	0.8037

**Fig 9:**
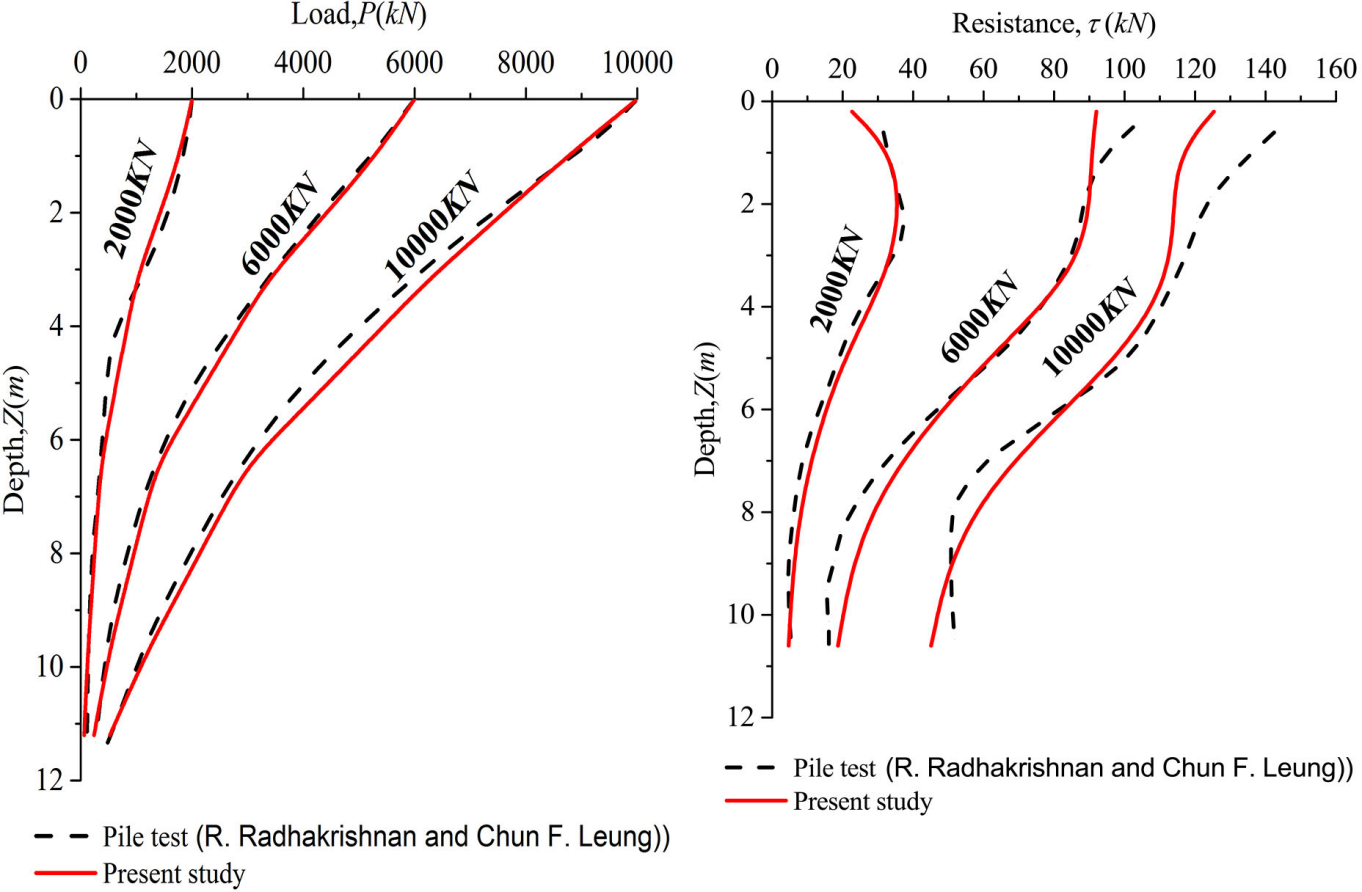
(a) Load distribution versus depth relationship; (b) side resistance versus depth relationship.

**Fig 10:**
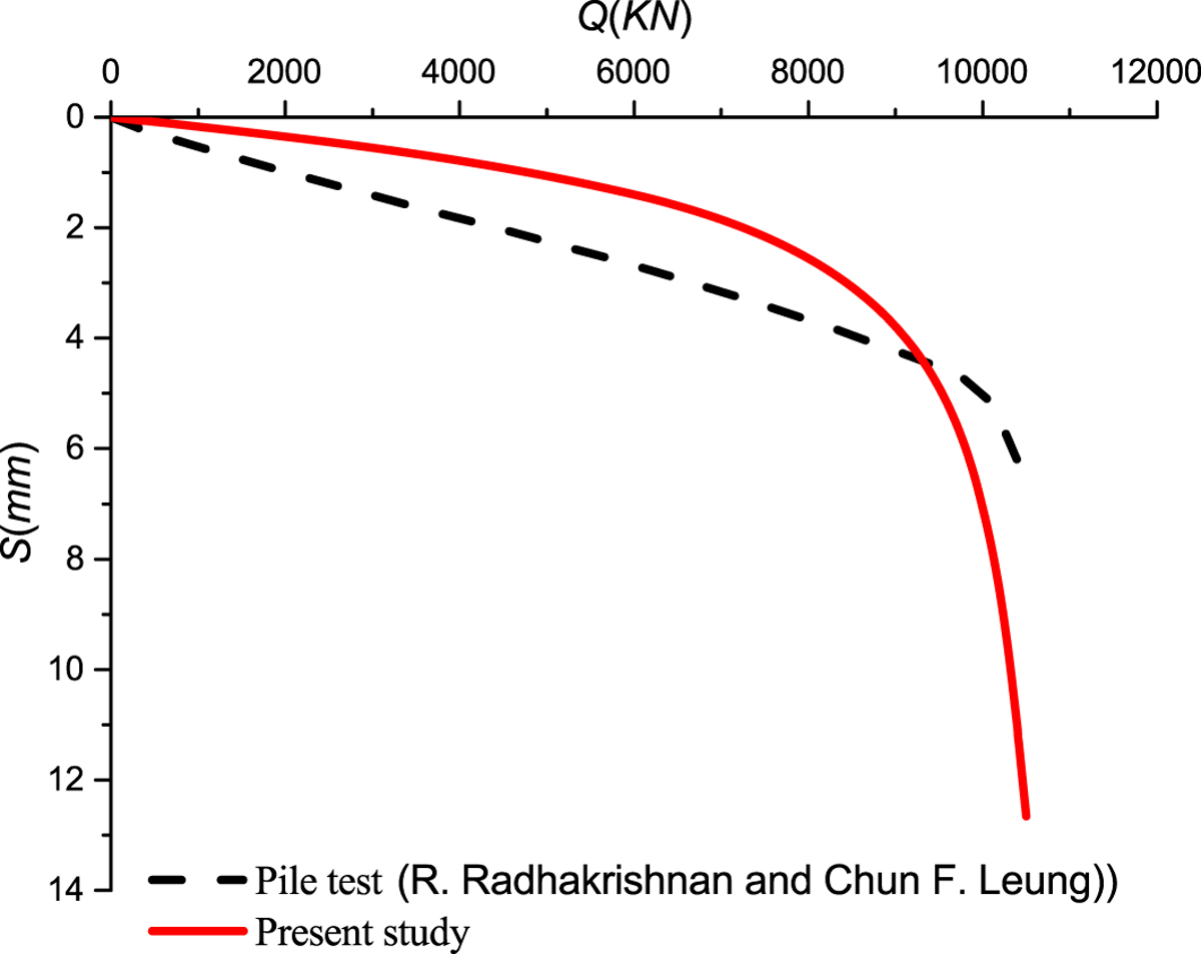
Load—settlement curves.

The load distribution and side resistance versus depth relationships are shown in Fig 9 for different vertical load. The predicted results are in good agreement with field tests in Fig 9A. However, the predicted side resistance is inconsistent with the measured result when *Q*≥6000*KN*. The small discrepancy may be attributable to the decreased shear displacement of the interface which inhibits the mobilization of side resistance of the pile and then induces that the *E*_*M*_ value of predicted result is higher than that of the measured result. As shown in Fig 10, a discrepancy from the comparison may be caused by the difference of side resistance between measured results and predicted results. Therefore, the realistic *Q*-*S* curve is below the predicted curve at the same settlement.

## Parametric study

The parameters of the crushing characteristics of crushed stones include lateral pressure coefficient, rolling load, relative breakage and the lithology of crushed stones. The effect of crushing characteristics on pile bearing capacity is analyzed with piles of 1.2m diameter, 14*m* depth in the crushing stones and 4.5*m* depth in the rock. The parameters for crushed stones are *α*_*s*_ = 2.0, *E*_*M*_ = 120*MPa*, *c* = 30*KPa* and *γ* = 20.7*KN/m*^3^. The parameters for the rock are *q*_*s*_ = 135*KPa*,*α*_*s*_ = 2.0 and *E*_*M*_ = 230*MPa*. The parameters for the pile base are *q*_*b*_ = 5000 *KPa*, *α*_*b*_ = 9.0, *E*_*M*_ = 330*MPa* and the Young’s modulus *E*_*b*_ = 31500*MPa*.

### Effect of lateral pressure coefficient

**Fig 11:**
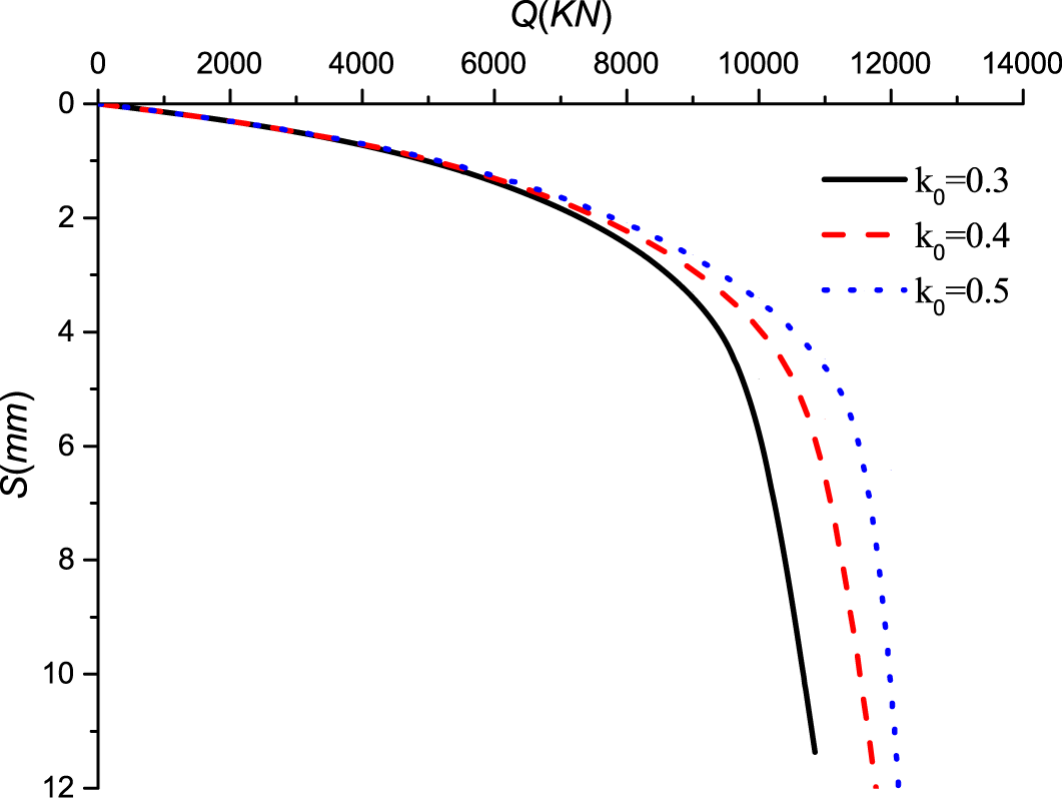
Effect of lateral pressure coefficient on *Q*-*S* curves.

**Fig 12:**
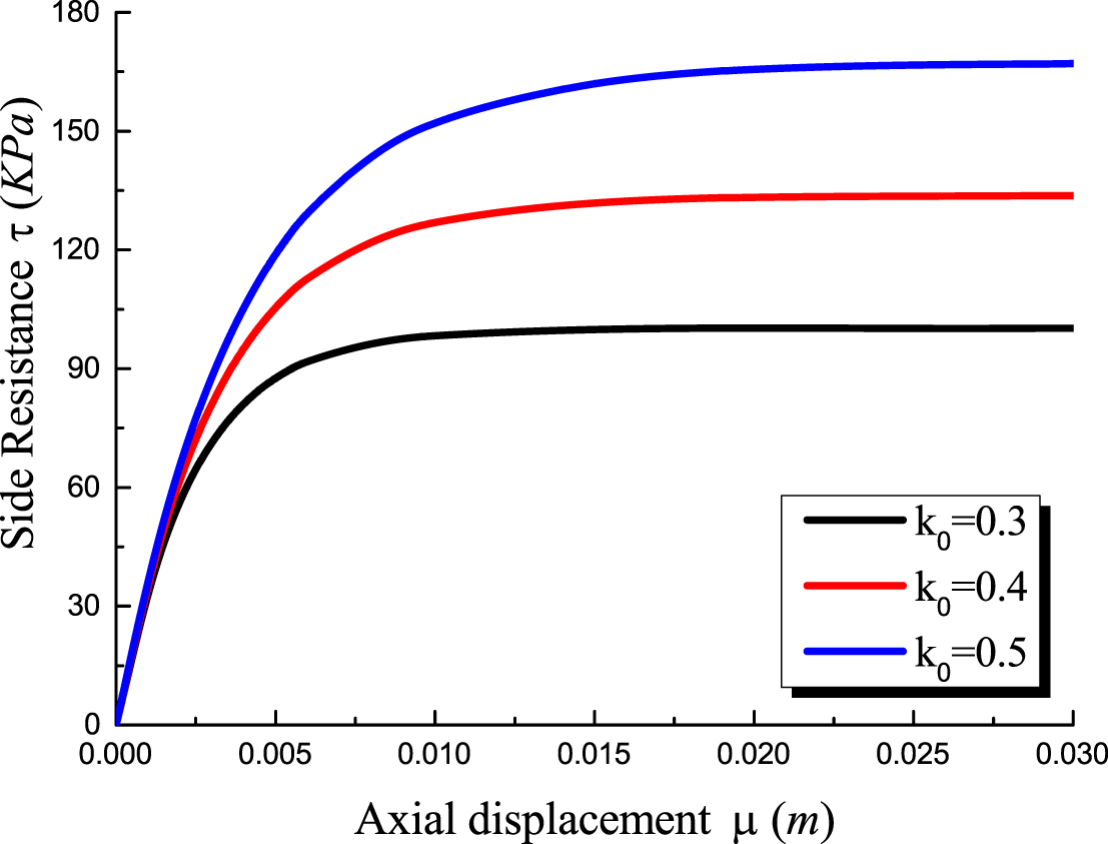
Effect of lateral pressure coefficient on *τ*-*μ* curves.

**Fig 13:**
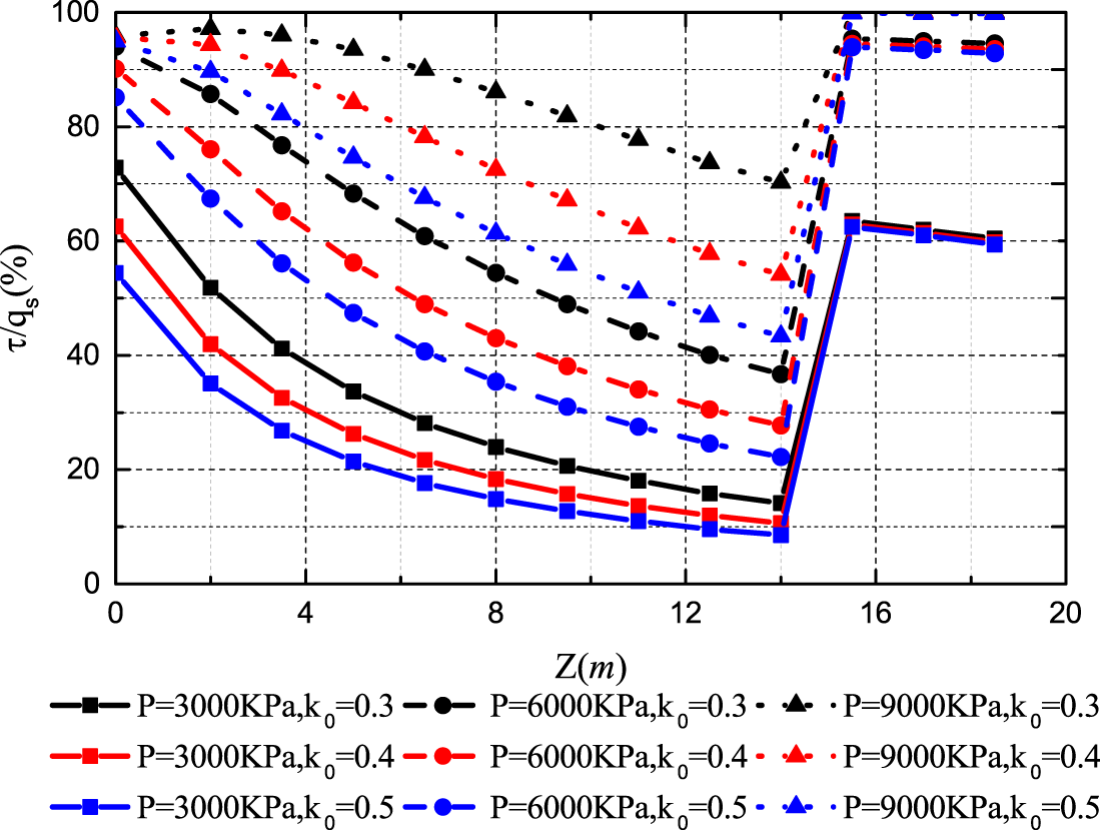
Effect of lateral pressure coefficient on *τ*/*q*_*s*_-*z* curves.

**Fig 14:**
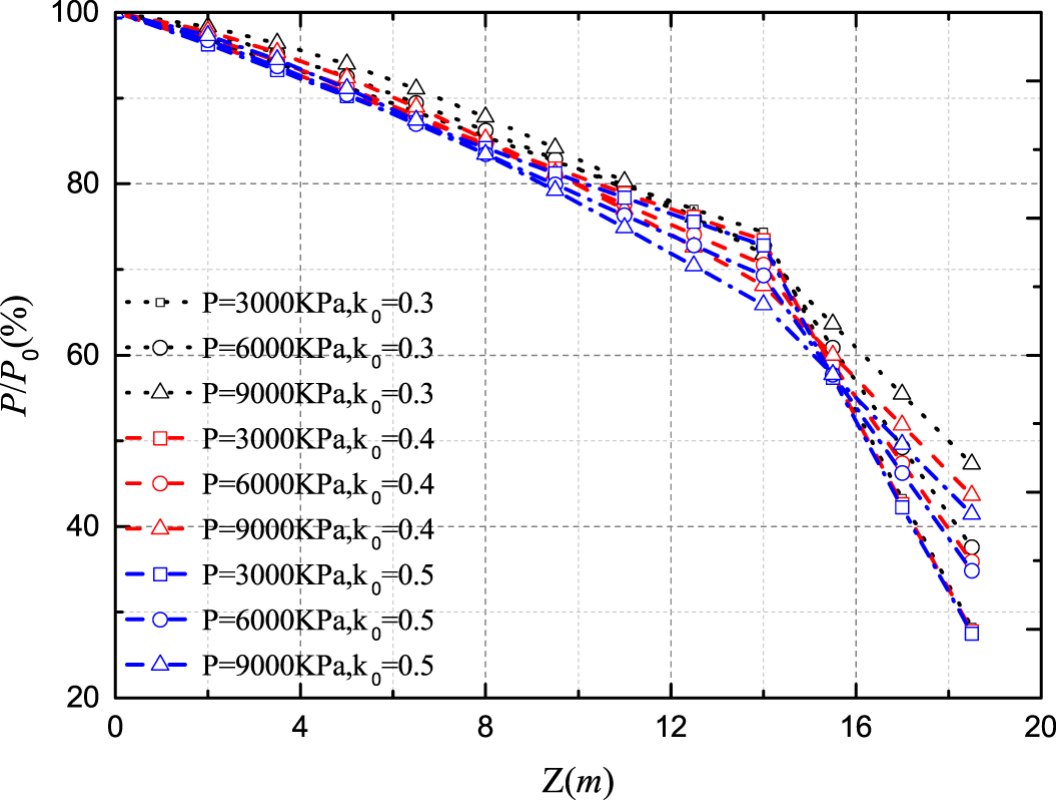
Effect of lateral pressure coefficient on *P*/*P*_*0*_-*z* curves.

The lateral pressure coefficient determines the mobilization of the horizontal stress around the pile. Figs 11–14 show the *Q-S* curves, *τ*-*μ* curves, *τ*/*q*_*s*_-*z* curves and *P*/*P*_*0*_-*z* curves of the pile in backfill area with different lateral pressure coefficient. The lithology of sample 2 is limestone and the rolling load is σ_3_ = 1500*KPa*.

As shown in Fig 11, with the increase of the lateral pressure coefficient, the ultimate bearing capacity of the pile becomes larger and the pile settlement decreases at the same load. When the ultimate bearing capacity of the pile is reached, the lateral pressure coefficient increases by 0.1 and the ultimate bearing capacity increases by 9.22% and 10.67%. Fig 12 shows that the increase of lateral pressure coefficient directly increases the ultimate side resistance. The lateral pressure coefficient increases by 0.1 and the ultimate side resistance increases by 33.33% and 33.33%. And the displacement *μ* increases when the ultimate side resistance is reached. In Figs 13 and 14, when the lateral pressure coefficient increases, the *τ*/*q*_*s*_ ratio and the *P*/*P*_*0*_ ratio decreases with the increase of pile depth *z*. When the *τ*/*q*_*s*_ ratio of the embedded rock section is stable, the *τ*/*q*_*s*_ ratio of the backfill section continues to increase. When the vertical load reaches 9000*KPa*, the lateral pressure coefficient increases by 0.1 and the *τ*/*q*_*s*_ ratio decreases by 22.98% and 15.31%, and the *P*/*P*_*0*_ ratio decreases by 5.07% and 3.12%.

### Effect of the rolling load and the relative breakage

**Fig 15:**
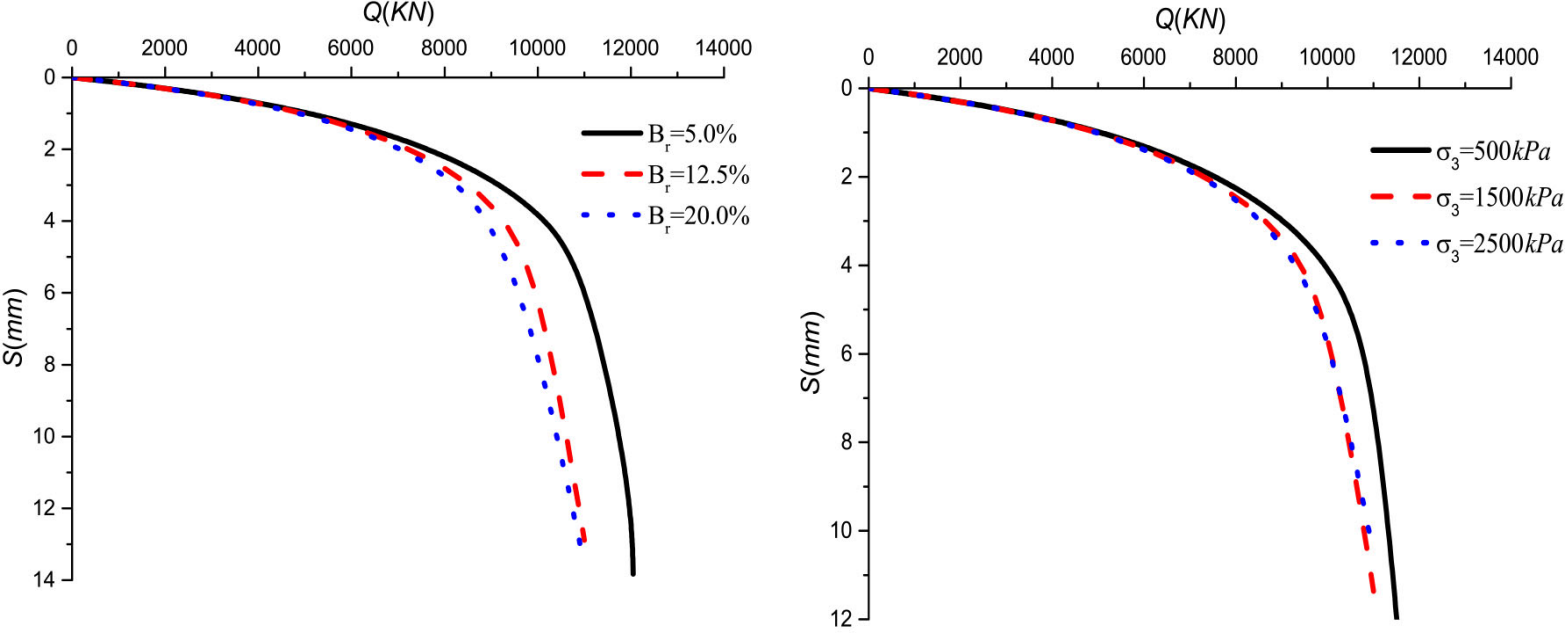
(a) Effect of the relative breakage on *Q*-*S* curves; (b) Effect of the rolling load on *Q*-*S* curves.

The relative breakage *B*_*r*_ of sample 2 was 5.0%, 12.5%, and 20.0%, respectively. The rolling load was 500*KPa*, 1500*KPa*, and 2500*KPa*. The lateral pressure coefficient is *k*_*0*_ = 0.4. Fig 15A and 15B show the *Q*-*S* curves of piles in crushed stones backfill area with the relative breakage of the crushed stones and the rolling load change, respectively.

**Fig 16:**
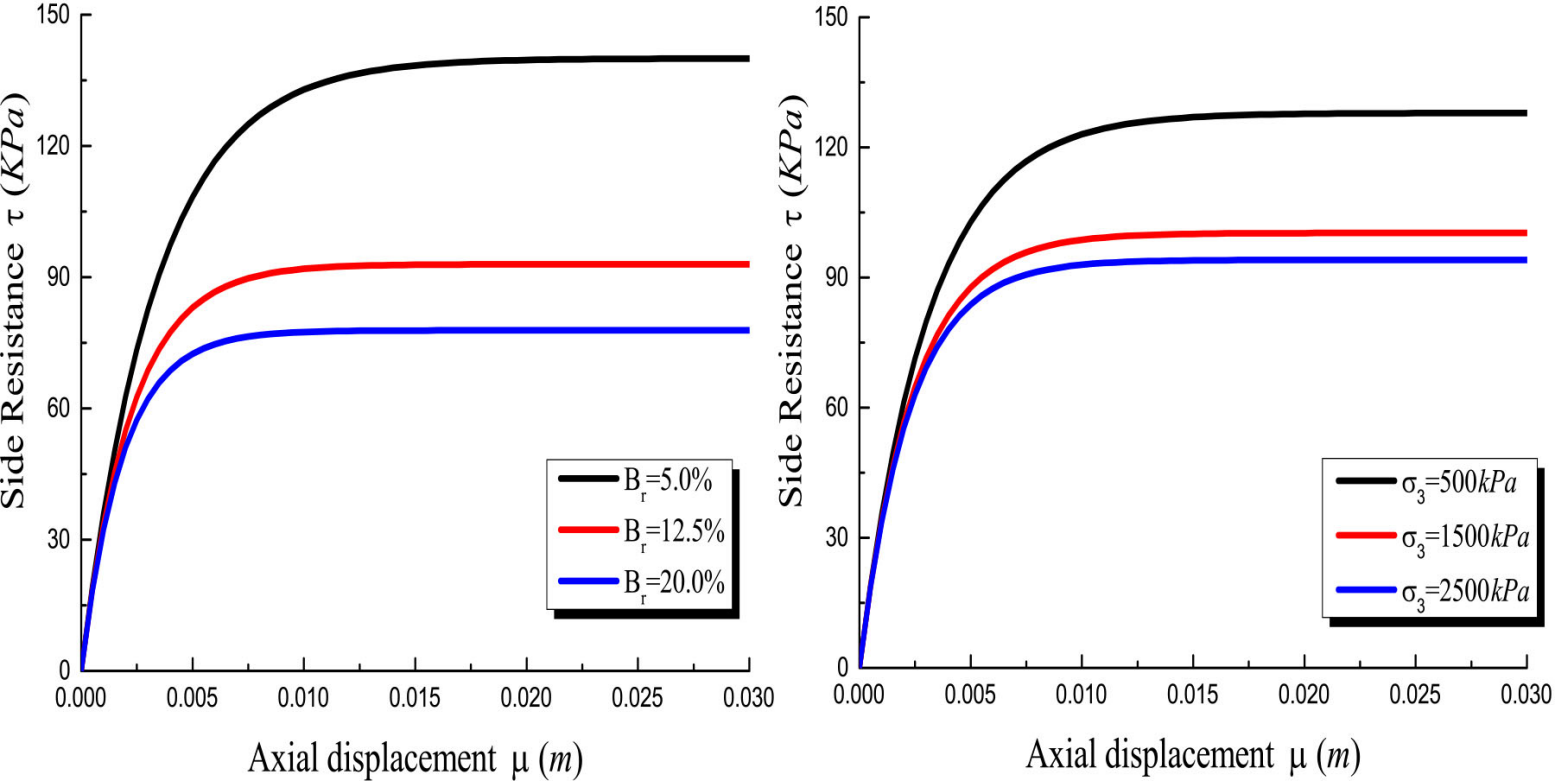
(a) Effect of the relative breakage on *τ*-*μ* curves; (b) Effect of the rolling load on *τ*-*μ* curves.

**Fig 17:**
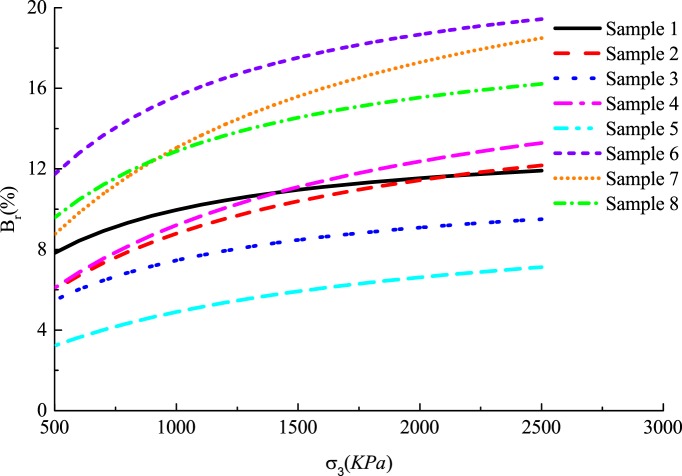
Relationship between the relative breakage and the rolling load.

It can be seen that the ultimate bearing capacity of the pile decreases with the increase of the rolling load and relative breakage in Fig 15. This trend becomes inconspicuous with the increase of the rolling load and the relative breakage. The decreases ratio by 10.63%, 4.8% in relative breakage, respectively, and the decreases ratio by 7.26%, 1.84% in the rolling load, respectively. This is because the increase in the relative breakage of the crushed stones reduces the pile side resistance. Fig 16 shows that the relative breakage increases by 7.5% and the ultimate side resistance decreases by 33.6% and 16.26%, and rolling load increases by 1000*KPa* and the ultimate side resistance decreases by 21.62% and 6.18%. As shown in Fig 17, the relative breakage increases and the growth rate becomes slow with the increase of the rolling load. Pile side resistance decreases as the relative breakage of crushed stones increases. In addition, the relative breakage of sample 1 and sample 2 are very close when the rolling load reaches 2500*KPa*.

**Fig 18:**
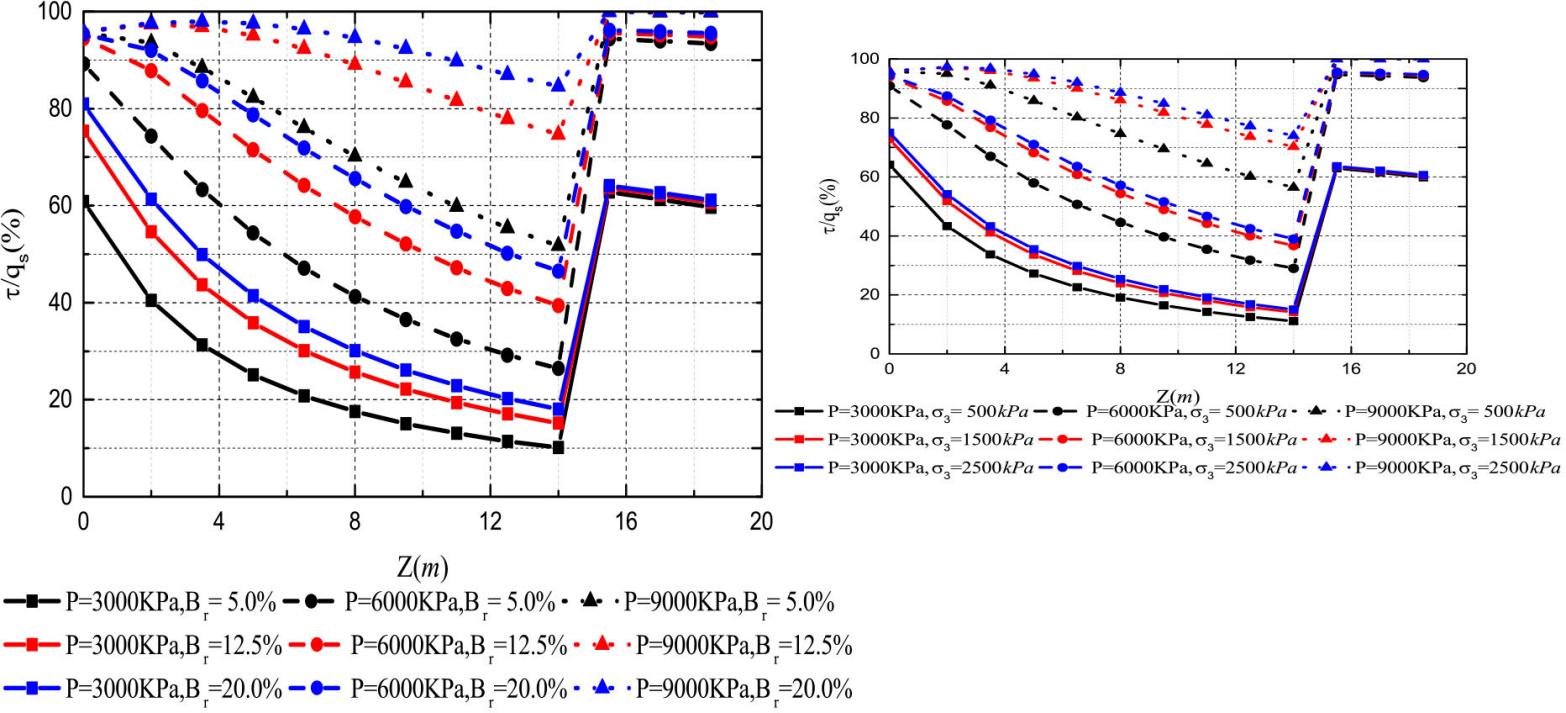
**(a) Effect of the relative breakage on *τ*/*q***_***s***_**-*z* curves; (b) Effect of the rolling load on *τ*/*q***_***s***_**-*z* curves**.

**Fig 19:**
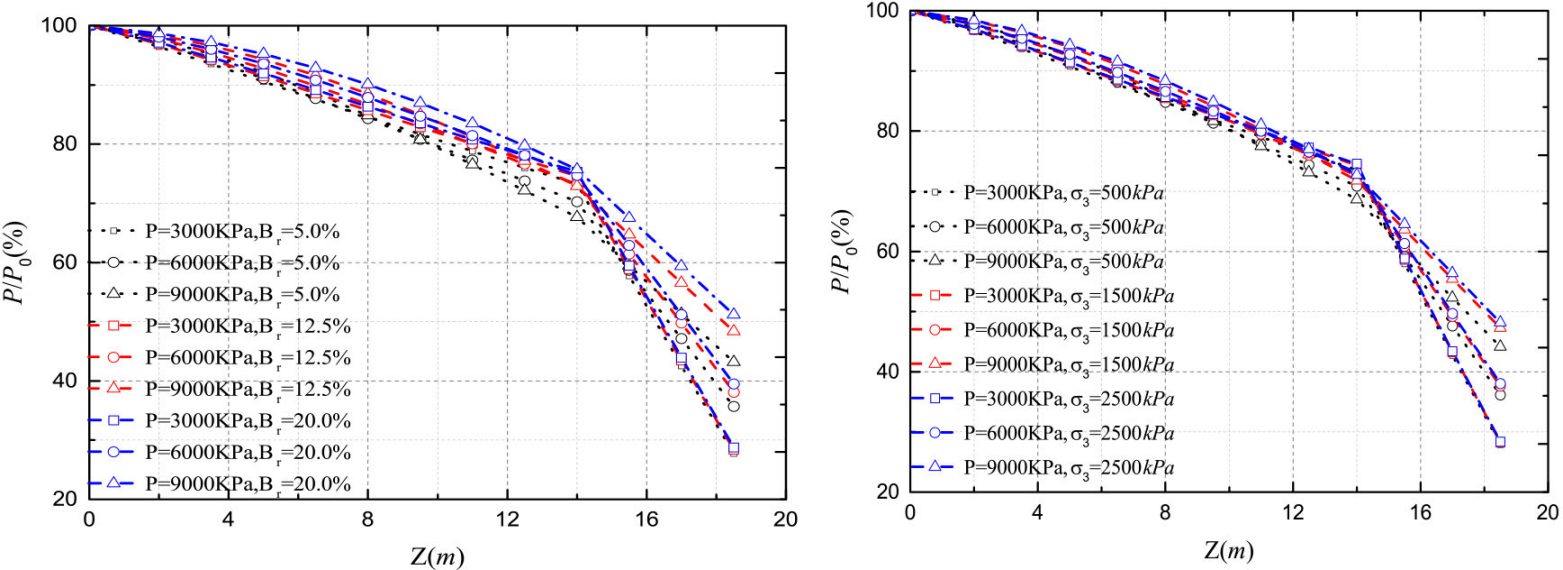
(a) Effect of the relative breakage on *P*/*P*_*0*_-*z* curves; (b) Effect of the rolling load on *P*/*P*_*0*_-*z* curves.

Figs 18 and 19 show that the side resistance increases gradually as the relative breakage and the rolling load increase. However, the effect of the relative breakage and the rolling load on the mobilization of the side resistance is gradually insignificant as the relative breakage and the rolling load increase. At the vertical load of 9000*KN*, the *τ*/*q*_*s*_ ratio increases initially and then decreases as the depth increases. Because the axial displacement decreases and the ultimate shear strength increases with the depth, the maximum side resistance is reached at a certain depth. Generally, the increase of the relative breakage and the rolling load promotes the mobilization of the side resistance, which makes the axial load shorn off more easily.

### Effect of crushed stones lithology

**Fig 20:**
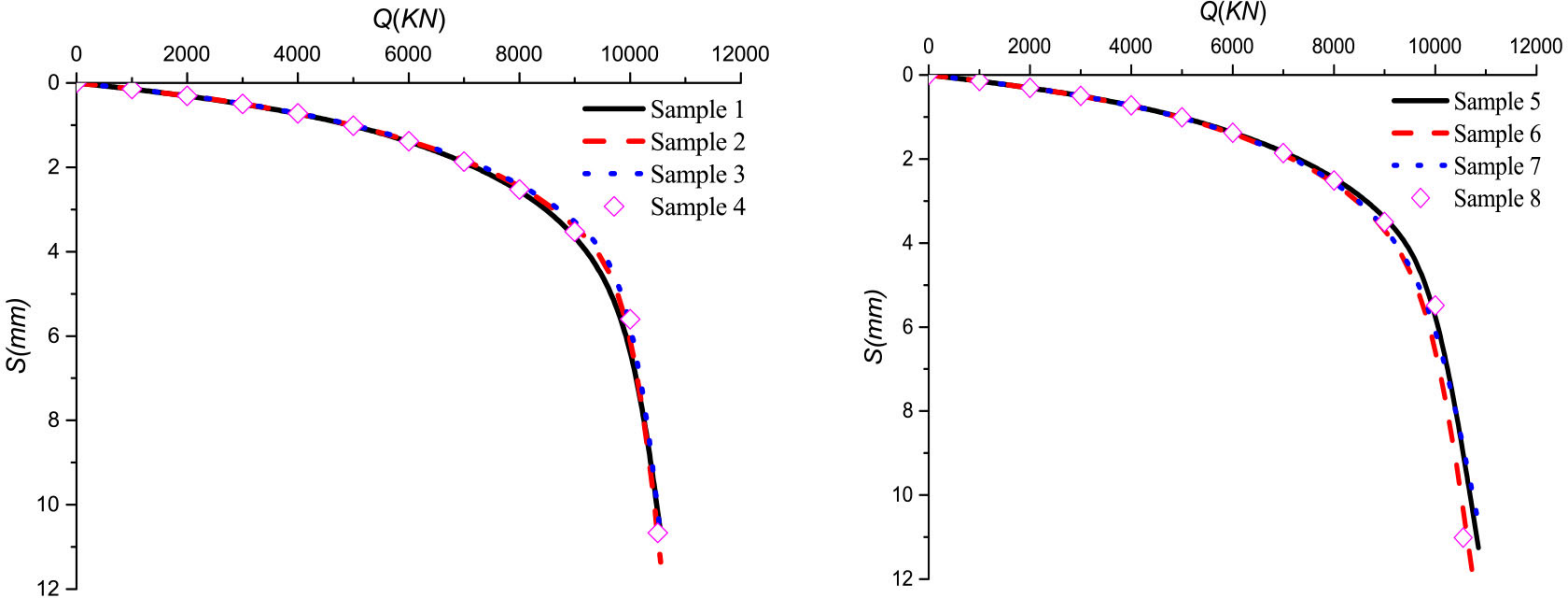
Effect of the samples on *Q*-*S* curves.

**Fig 21:**
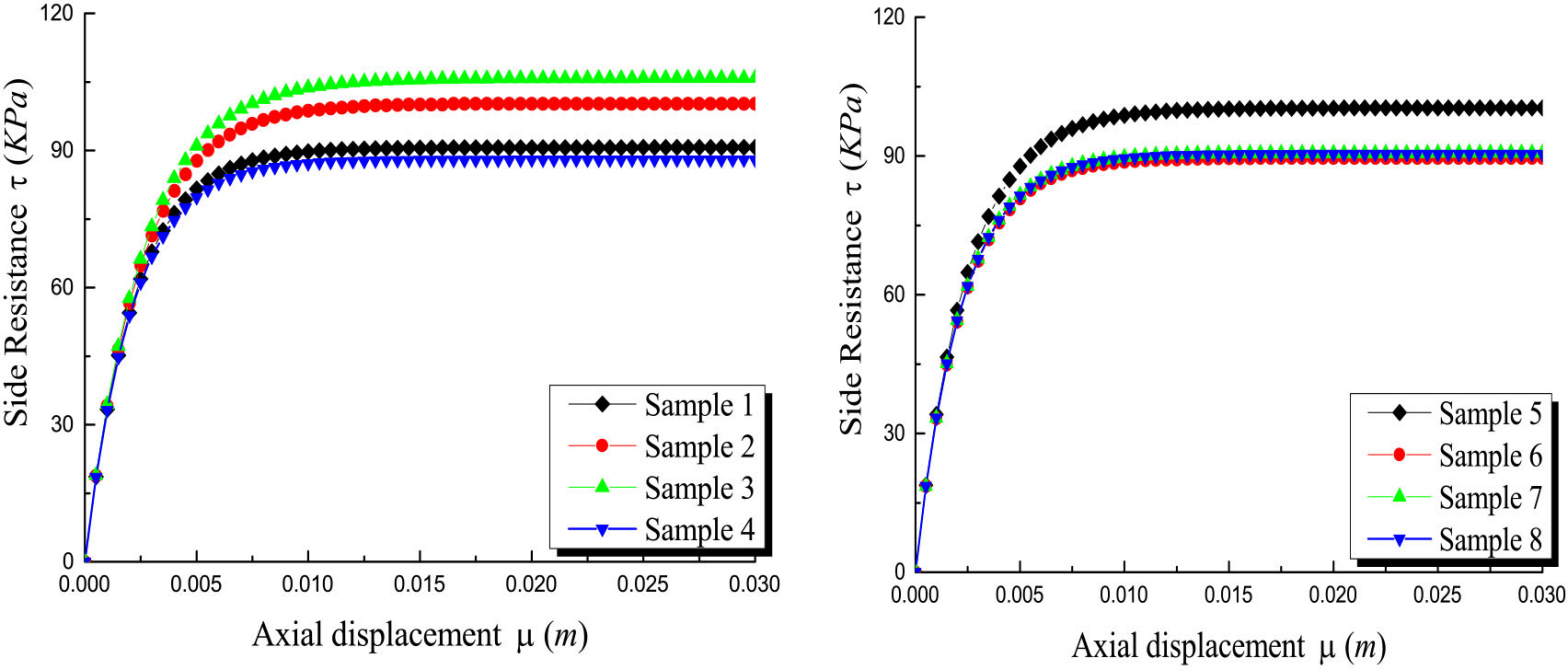
Effect of the samples on *τ*-*μ* curves.

**Fig 22:**
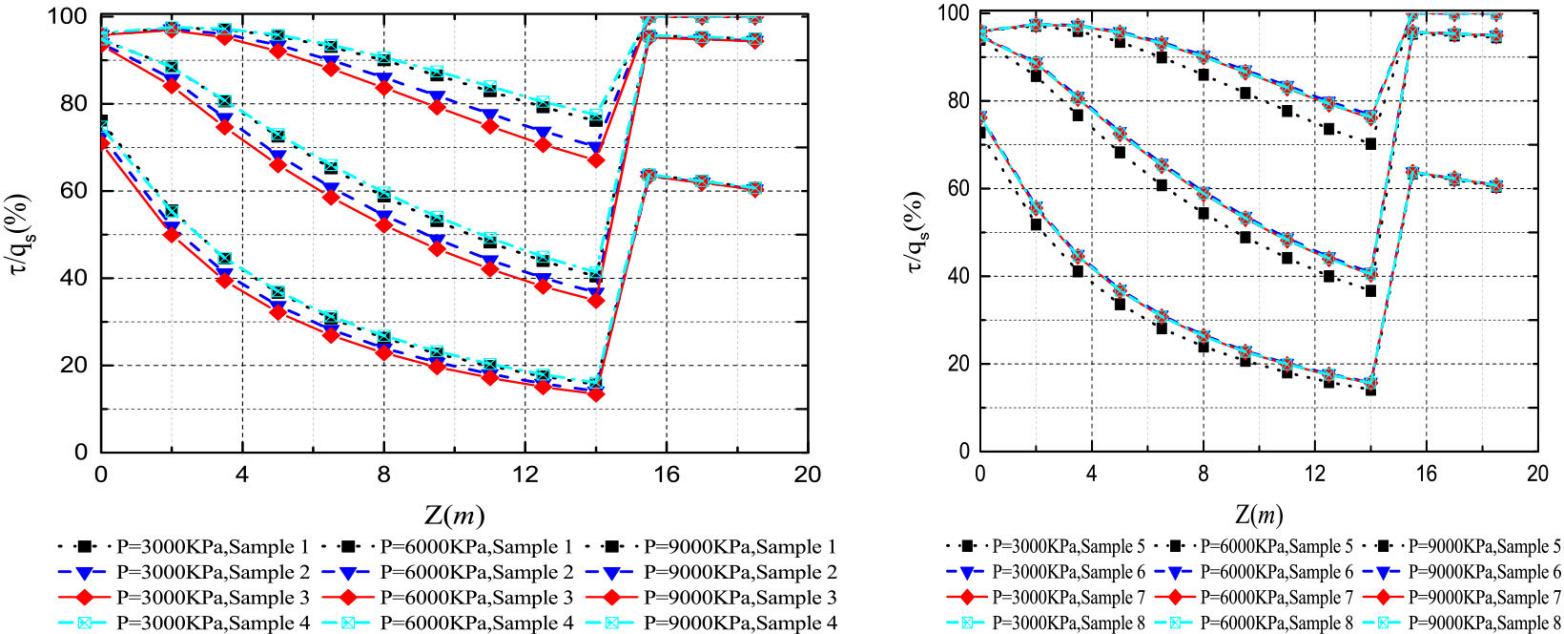
Effect of the samples on *τ*/*q*_*s*_-*z* curves.

**Fig 23:**
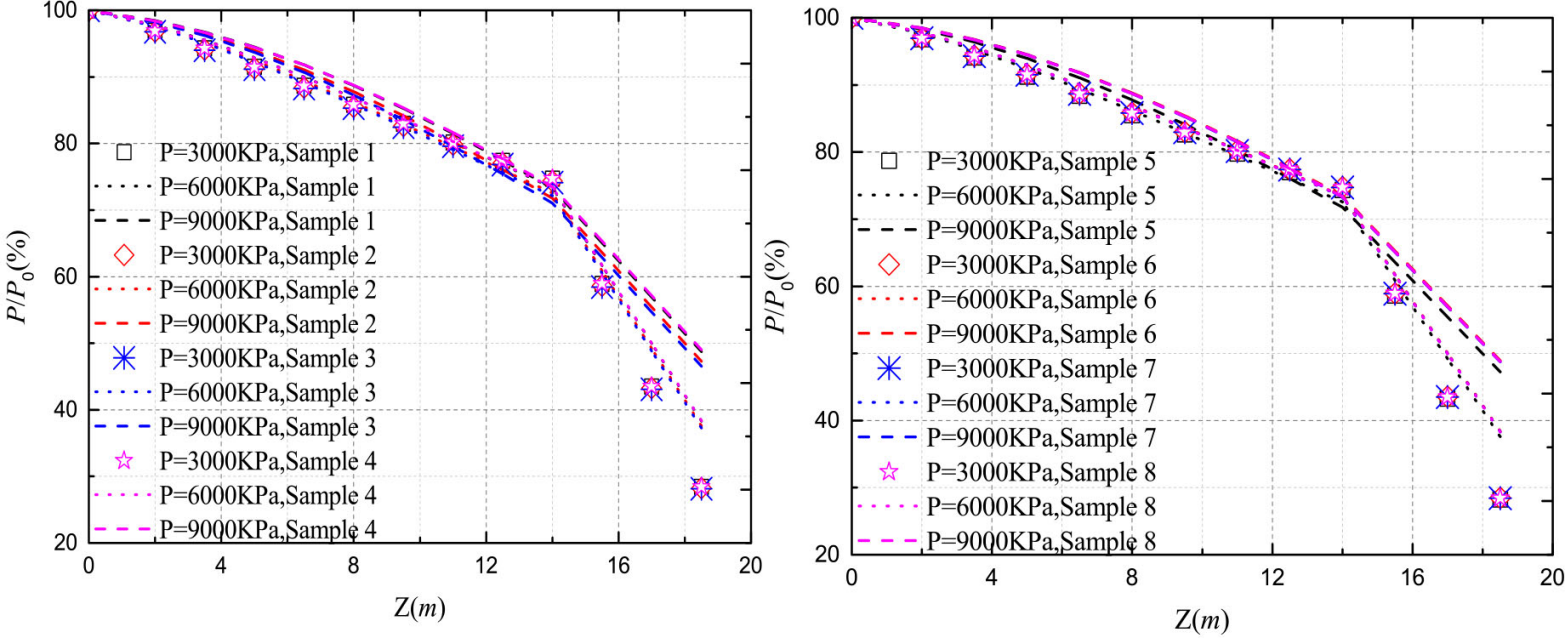
Effect of the samples on *P*/*P*_*0*_-*z* curves.

Figs 20–23 show the *Q*-*S* curves, *τ*-*μ* curves, τ/q_s_-z curves, and P/P_0_-z curves of the pile in backfill area with different crushed stones lithology, respectively. The lithologies for eight samples are quartz sandstone, Limestone et.al. The lateral pressure coefficient is 0.4 and the rolling load is 1500*KPa*.

It is obvious that there is little difference between the eight samples and the ultimate load of the pile is about 10600*KN* in Fig 20. As shown in Fig 21, the ultimate side resistance of the eight samples ranged from 90*KPa* to 105*KPa* and the ratio of the length of the interval to the maximum ultimate side resistance is 0.143. It illustrates that the effect of different samples on the ultimate side resistance is very small. Because the relative breakage and the peak internal friction angle curve in the literature [31] have little change in lithology, the ultimate side resistance is less affected by lithology. From Figs 22 and 23, it can be seen that the *τ*/*q*_*s*_ ratio and the *P*/*P*_*0*_ ratio are not changed by the lithology of the crushed stones.

## Conclusion

A load transfer model for the rock-socketed pile considering the relationship between the crushing characteristics and the nonlinear load transfer function was built in the large karst cave. Using the finite difference method, the transfer equation of load function was derived. In addition, the effect of the key parameters including the lateral pressure coefficient, the relative breakage, the rolling load and the crushed stones lithology on the bearing mechanism of the rock-socketed pile in the crushed stones backfill was analyzed. Main conclusions can be summarized as follows.

1. A load transfer function model was built to reflect the load-settlement behavior of the rock-socketed pile in the crushed stones backfill area. The proposed model was verified by the comparison of two cases.
2. The lateral pressure coefficient has a significant effect on the ultimate bearing capacity and the mobilization of side resistance of pile. The ultimate side resistance and the ultimate bearing capacity gradually increase as the lateral pressure coefficient increases. The side resistance increases initially and then decreases as the depth increases at high load levels.
3. The increase in relative breakage and the rolling load increases the ultimate bearing capacity of the pile and the mobilization of side resistance, this trend becomes not obvious with the increase of the rolling load and the relative breakage. At the vertical load of 9000KN, the *τ*/*q*_*s*_ ratio increases initially and then decreases as the depth increases. The crushed stones lithology has little effect on the ultimate bearing capacity (about 10600*KN*) and the ultimate side resistance (ranging from 90*KPa* to 105*KPa*) by the comparison of eight samples. Thus, to improve the pile bearing capacity, the relative breakage of crushed stones should be reduced.
4. By analyzing the *Q*-*S* curves, *τ*-*μ*curves, *τ*/*q*_*s*_-z curves, and *P*/*P*_*0*_-z curves, it can be concluded that the side resistance of the pile in the crushed stones backfill area cannot be ignored in term of avoiding the waste of engineering materials. If the test parameters of the crushed stones and the rolling load are obtained, the relative breakage and the bearing capacity of pile can be estimated. The present study proposes a method to assess the vertically loaded pile response considering crushing characteristics. The analysis can be further extended to account for characteristics of crushed stones and to study on combined loads.

## Supporting information

S1 File Data of all figures. (ZIP)

## Nomenclature

q_s_: the ultimate side resistance
q_b_: the ultimate base resistance
μ: the axial displacement
μ_b_: the base axial displacement
τ: the side resistance
q: the base resistance
E_M_: the Ménard modulus
B: the pile diameter
B_r_: the relative breakage
σ_3_: the rolling load
n: the number of segments of pile
φ_p_: the peak internal friction angle
c: the cohesion of crushed stones
σ: the horizontal stresses
k_0_: the lateral pressure coefficient
γ: the crushed stones bulk density
z: The depth of calculated point
P_a_: the atmospheric pressure
E_i_: the Young’s modulus
A_i_: the section area of the pile
U: the circumference of pile
L: the pile length
h: the length of each of segment
$\mu^{'}{}_{i}$: the derivative of *μ*_*i*_
μ_i_: the displacement of the i-th node
df: a differential of “percent passing” divided by 100
b_p_: the potential for breakage that is significant to soil behavior
*a*,*b*,*j*,*k*: the fitting parameters of the particle
b_p0_: the original values of *b*_*p*_
b_pl_: the original values of *b*_*p*_ after loading
*μ*_ib_: the base displacement of i-th node
μ_it_: the top displacement of i-th node
α_s_: the factor taking into account the influence of the soil type
α_b_: the factor taking into account the influence of the rock type
k_s_: the initial stiffness of side resistance
k_b_: the initial stiffness of base resistance
λ_s_: the parameter taking into account the influence of the crushed stones type
λ_b_: the parameter taking into account the influence of the rock type
*τ*(*μ*): the function describing the variation of the mobilized side resistance *τ* in function of the axial displacement *μ*
*q*(*μ*_*b*_): the function describing the variation of the mobilized base resistance *q* in function of the axial displacement *μ*_*b*_
